# Supplementary material for: A macroevolutionary common-garden experiment reveals differentially evolvable bone organization levels in slow arboreal mammals
Source: Commun Biol. 2023 Sep 28;6:995. doi: 10.1038/s42003-023-05371-3 (PMC10539518; doi:10.1038/s42003-023-05371-3)
Supplement: Supplementary file 2 — Supplementary Information [file 42003_2023_5371_MOESM2_ESM.pdf]

A macroevolutionary common-garden experiment reveals differentially evolvable bone organization levels in slow arboreal mammals

Fabio Alfieri[1,2], Léo Botton-Divet[1], Jan Wölfer[1], John A. Nyakatura[1], Eli Amson[3]

1. Comparative Zoology, Institute for Biology, Humboldt-Universität zu Berlin, Unter den Linden 6, 10117 Berlin, Germany
2. Museum Für Naturkunde, Leibniz-Institut für Evolutions- und Biodiversitätsforschung, Invalidenstraße 43 10115 Berlin, Germany
3. Paleontology Department, Staatliches Museum für Naturkunde, Rosenstein 1-3, 70191 Stuttgart, Germany

Corresponding author: Fabio Alfieri, email: fabio_alfieri@yahoo.it

ORCID:

Fabio Alfieri: 0000-0001-8241-7309

Léo Botton-Divet: 0000-0002-3931-2752

Jan Wölfer: 0000-0001-8630-2461

John A. Nyakatura: 0000-0001-8088-8684

Eli Amson: 0000-0003-1474-9613

Supplementary Information

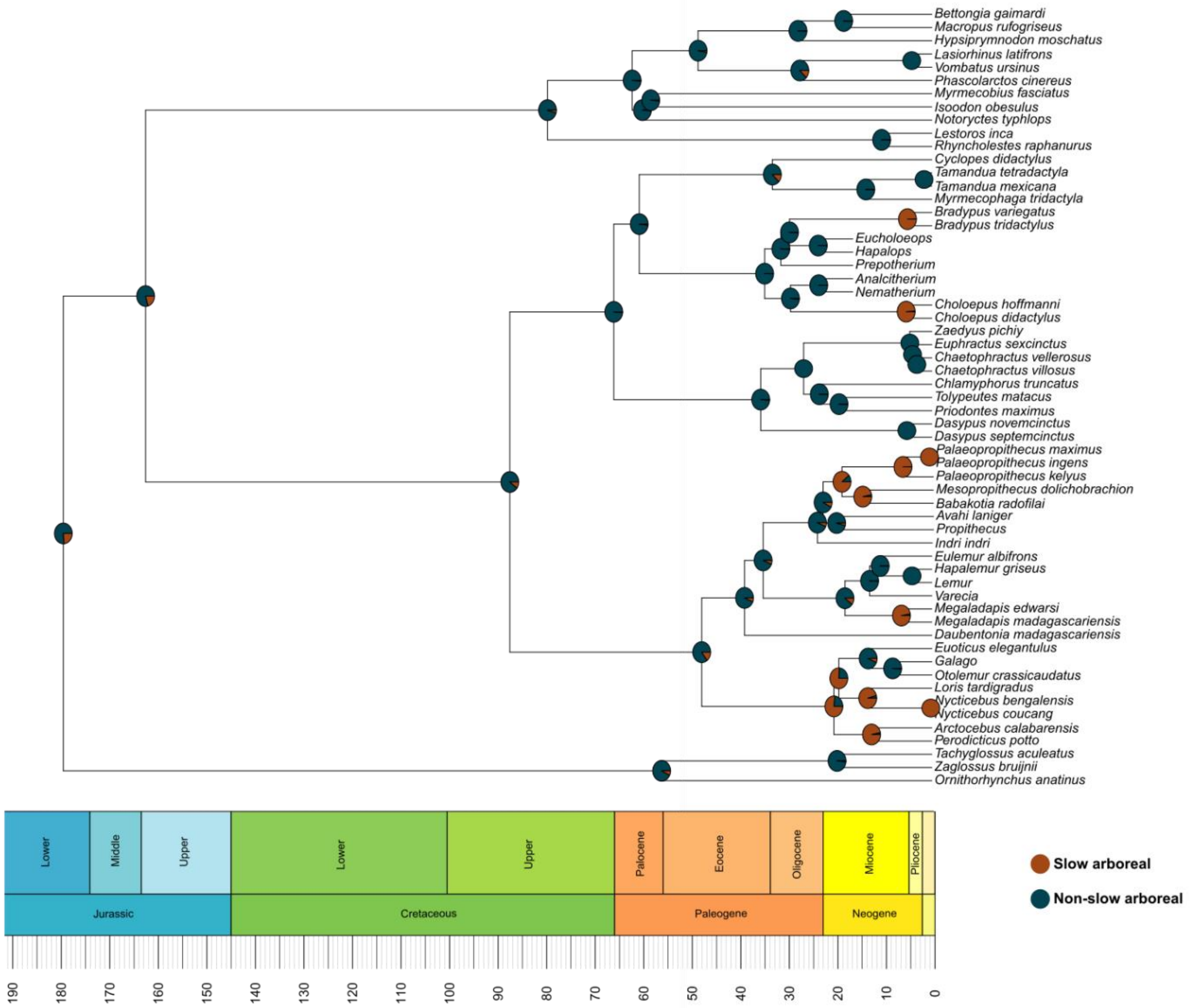

Supplementary Fig. 1. Time-calibrated phylogeny used to perform Ancestral Lifestyle Reconstruction. Twelve additional taxa (not shown in Fig. 2) were included. On pie charts on internal nodes the most likely ancestral lifestyle reconstruction is showed.

Supplementary Table 1

Common name	Scientific name	Lifestyle	Reference(s)
Wombats	*Lasiorhinus latifrons*<br>*Vombatus ursinus*	**Non-slow arboreal**	[1]
Tamanduas	*Tamandua* spp.	**Non-slow arboreal**	[2]
Giant anteater	*Myrmecophaga tridactyla*	**Non-slow arboreal**	[2,3]
Medium-small body sized ground sloths	*Eucholoeops, Hapalops, Nematherium, Analcitherium Prepotherium*	**Non-slow arboreal**	[4,5]
Armadillos	Cingulata	**Non-slow arboreal**	[6,7]
Indriids	'Indriidae'	**Non-slow arboreal**	[8–10]
Lemurids	Lemuridae	**Non-slow arboreal**	[10,11]
Galagos	Galagidae	**Non-slow arboreal**	[9,10,12]
Three-toed sloths	*Bradypus* spp.	**Slow arboreal**	[13–16]
Two-toed sloths	*Choloepus* spp.	**Slow arboreal**	[13–16]
Silky anteater	*Cyclopes didactylus*	**Slow arboreal**	[17–19]
Koala lemurs	*Megaladapis* spp.	**Slow arboreal**	[20,21]
Sloth lemurs	Palaeopropithecidae	**Slow arboreal**	[20,21]
Koala	*Phascolarctos cinereus*	**Slow arboreal**	[1,22,23]
Lorisids	'Lorisidae'	**Slow arboreal**	[24–26]

References in Supplementary Table 1

1. Tyndale-Biscoe, C. H. *Life of Marsupials*. (Csiro Publishing (doi: 10.1071/9780643092204), 2005).
2. Amson, E., Arnold, P., van Heteren, A. H., Canoville, A. & Nyakatura, J. A. Trabecular architecture in the forelimb epiphyses of extant xenarthrans (Mammalia). *Front Zool* **14**, 52 (2017).
3. Orr, C. M. Knuckle-walking anteater: a convergence test of adaptation for purported knuckle-walking features of African Hominidae. *Am. J. Phys. Anthropol.* **128**, 639–658 (2005).
4. Toledo, N. Paleobiological integration of Santacrucian Sloths (Early Miocene of Patagonia). *Ameghiniana* **53**, 100 (2016).
5. Alfieri, F., Botton-Divet, L., Nyakatura, J. A. & Amson, E. Integrative approach uncovers new patterns of ecomorphological convergence in slow arboreal xenarthrans. *J Mamm Evol* (2022) doi:10.1007/s10914-021-09590-5.
6. Vizcaíno, S. F. & Milne, N. Structure and function in armadillo limbs (Mammalia: Xenarthra: Dasypodidae). *J. Zool.* **257**, 117–127 (2002).
7. Attias, N., Miranda, F. R., Sena, L. M. M., Tomas, W. M. & Mourão, G. M. Yes, they can! Three-banded armadillos *Tolypeutes* sp. (Cingulata: Dasypodidae) dig their own burrows. *Zoologia (Curitiba)* **33**, e20160035 (2016).
8. Napier, J. R. & Walker, A. C. Vertical clinging and leaping-a newly recognized category of locomotor behaviour of primates. *Folia Primatol.* **6**, 204–219 (1967).
9. Gebo, D. L. Vertical clinging and leaping revisited: Vertical support use as the ancestral condition of strepsirrhine primates. *Am. J. Phys. Anthropol.* **146**, 323–335 (2011).
10. Fleagle, J. G. *Primate Adaptation and Evolution - 3rd Edition*. (Academic press. (doi:10.1016/C2009-0-01979-5), 2013).
11. Hill, W. C. O. *Primates: Comparative Anatomy and Taxonomy: Vol. 1. Strepsirhini.* (Edinburgh University Press. (doi: 10.1002/ajpa.1330140411), 1953).

12. Walker, A. Prosimian Locomotor Behavior. in *In: The Study of Prosimian Behavior.* 543–565 (Doyle GA. and Martin RD. (doi: 10.1016/B978-0-12-222150-7.X5001-6), 1979).
13. Britton, S. W. & Kline, R. F. Augmentation of activity in the sloth by adrenal extract, emotion and other conditions. *Am. J. Physiol.* **127**, 127–130 (1939).
14. Brattstrom, B. H. Sloth behavior. *Journal of Mammalogy* **47**, 348–348 (1966).
15. Pauli, J. N., Peery, M. Z., Fountain, E. D. & Karasov, W. H. Arboreal folivores limit their energetic output, all the way to slothfulness. *Am. Nat.* **188**, 196–204 (2016).
16. Vendl, C. *et al.* Digestive physiology, metabolism and methane production of captive Linné's two-toed sloths (*Choloepus didactylus*). *J Anim Physiol Anim Nutr (Berl)* **100**, 552–564 (2016).
17. van Tyne, J. Notes on the habits of *Cyclopes dorsalis*. *J. Mammal.* **10**, 314 (1929).
18. Hayssen, V., Miranda, F. & Pasch, B. *Cyclopes didactylus* (Pilosa: Cyclopedidae). *Mamm. Species* **44**, 51–58 (2012).
19. Nagy, K. A. & Montgomery, G. G. Field metabolic rate, water flux and food consumption by free-living silky anteaters (*Cyclopes didactylus*) in Panama. *Edentata* **13**, 61–65 (2012).
20. Godfrey, L. R., Jungers, W. L. & Schwartz, G. T. Ecology and Extinction of Madagascar's Subfossil Lemurs. in *Lemurs: Ecology and Adaptation* 41–64 (Gould L., Sauther M.L. (doi: 10.1007/978-0-387-34586-4_3), 2006).
21. Godfrey, L. R., Granatosky, M. C. & Jungers, W. L. The Hands of Subfossil Lemurs. in *In: The Evolution of the Primate Hand. Anatomical, Developmental, Functional, and Paleontological Evidence.* 421–453 (Kivell T.L., Lemelin P., Richmond B.G., Schmitt D. (doi: 10.1007/978-1-4939-3646-5_15), 2016).
22. Nagy, K. A. & Martin, R. W. Field metabolic rate, water flux, food consumption and time budget of koalas, *Phascolarctos cinereus* (Marsupialia: Phascolarctidae) in Victoria. *Aust. J. Zool.* **33**, 655–665 (1985).
23. Grand, T. I. & Barboza, P. S. Anatomy and development of the koala, *Phascolarctos cinereus*: an evolutionary perspective on the superfamily Vombatoidea. *Anat Embryol* **203**, 211–223 (2001).
24. Walker, A. The Locomotion of the lorises, with special reference to the potto. *Afr. J. Ecol.* **7**, 1–5 (1969).
25. Rasmussen, D. T. & Izard, M. K. Scaling of growth and life history traits relative to body size, brain size, and metabolic rate in Lorises and Galagos (Lorisidae, primates). *Am. J. Phys. Anthropol.* **75**, 357–367 (1988).
26. Jouffroy, F. & Petter, A. Gravity-related kinematic changes in lorisine horizontal locomotion in relation to position of the body. in *Gravity, Posture and Locomotion in Primates.* 199–208 (Jouffroy F., Stack M. and Niemitz C. (doi:10.1007/BF02547675), 1990).

Supplementary Table 2. Summary statistics of PGLSs (value, standard error, t-value and p-value) performed on humeral traits. BM is the abbreviation for Body Mass. Sample sizes for external shape analyses: n slow arboreal species= 17; n non-slow arboreal species=26; Sample sizes for mid-diaphyseal structure analyses: n slow arboreal species= 17; n non-slow arboreal species=27; Sample sizes for average diaphyseal structure analyses: n slow arboreal species= 15; n non-slow arboreal species=26; Sample sizes for proximal epiphyseal trabecular structure analyses: n slow arboreal species= 15; n non-slow arboreal species=26; Sample sizes for distal epiphyseal trabecular structure analyses: n slow arboreal species: 16; n non-slow arboreal species=25.

Variable	Lifestyle.value	Lifestyle.Std.Err	Lifestyle.t.value	Lifestyle.p.value	BM. value	BM. Std.Err	BM t.val	BM p.val	method	λ
**3D GM PC1**	-0,038	0,020	-1,858	0,071	0.005	0.017	0.271	0.788	ML	0.999
**3D GM PC2**	0,005	0,007	0,750	0,458	-0.009	0.006	-1.599	0.118	ML	0.996
**3D GM PC3**	-0,005	0,012	-0,422	0,675	0.021	0.010	2.023	0.050	ML	1
**3D GM PC4**	-0,028	0,006	-4,785	0,000	0.017	0.005	3.509	0.001	REML	1
**3D GM PC5**	-0,003	0,006	-0,553	0,583	0.022	0.006	4.019	0.000	ML	0.928
**3D GM PC6**	-0,005	0,006	-0,837	0,408	0.000	0.005	-0.044	0.965	ML	0
**3D GM PC7**	0,003	0,007	0,380	0,706	0.001	0.000	3558.457	0.000	ML	1
**3D GM PC8**	0,001	0,004	0,178	0,860	-0.004	0.003	-1.070	0.291	REML	0
**3D GM PC9**	0,006	0,004	1,702	0,097	0.005	0.003	1.435	0.159	REML	0.914
**3D GM PC10**	0,003	0,004	0,700	0,488	-0.002	0.003	-0.483	0.632	REML	0
**Log-ResC50**	0,098	0,039	2,503	0,016	0.070	0.036	1.942	0.059	ML	0.551
**Log-ResCAver**	0,105	0,038	2,762	0,009	0.077	0.034	2.290	0.028	ML	0.486
**Log-Imax50**	-0,723	0,416	-1,738	0,090	4.187	0.377	11.107	0.000	ML	0.971
**Log-ImaxAver**	-0,864	0,496	-1,742	0,090	3.928	0.398	9.871	0.000	ML	1
**Log-Imin50**	-0,707	0,334	-2,120	0,040	4.172	0.317	13.174	0.000	ML	0.87
**Log-IminAver**	-0,859	0,352	-2,443	0,019	3.937	0.323	12.206	0.000	ML	0.824
**Log-CSA50**	-0,272	0,186	-1,467	0,150	2.137	0.173	12.380	0.000	ML	0.94
**Log-CSAAver**	-0,351	0,194	-1,808	0,078	2.046	0.177	11.583	0.000	ML	0.892
**Log-CSS50**	-0,068	0,154	-0,444	0,660	0.092	0.126	0.734	0.467	ML	1
**Log-CSSAver**	-0,036	0,085	-0,420	0,677	0.072	0.070	1.033	0.308	ML	0.998
**DAprox**	-0,076	0,027	-2,828	0,007	-0.069	0.027	-2.585	0.014	ML	0.708
**Log-Tb.Th.$_{prox}$**	-0,023	0,067	-0,341	0,735	0.605	0.067	9.049	0.000	ML	0.739
**Log-Conn.D.$_{prox}$**	0,957	0,159	6,026	0,000	-1.722	0.142	-12.111	0.000	ML	0
**BV.TV.$_{prox}$**	-0,040	0,020	-1,946	0,059	0.058	0.018	3.296	0.002	REML	0.124
**Log-BS.TV.$_{prox}$**	0,070	0,060	1,168	0,250	-0.557	0.050	-11.108	0.000	REML	0
**Log-Av.Br.Len.$_{prox}$**	-0,176	0,053	-3,332	0,002	0.620	0.045	13.789	0.000	REML	0.088
**DAdist**	-0,117	0,030	-3,942	0,000	-0.073	0.032	-2.290	0.028	ML	0.778
**Log-Tb.Th.$_{dist}$**	0,016	0,080	0,204	0,839	0.598	0.085	7.001	0.000	ML	0.901
**Log-Conn.D.$_{dist}$**	0,294	0,161	1,828	0,075	-1.217	0.171	-7.116	0.000	REML	0.738
**Log-BV.TV.$_{dist}$**	-0,045	0,073	-0,620	0,539	0.138	0.078	1.774	0.084	ML	0.801
**Log-BS.TV.$_{dist}$**	0,007	0,060	0,122	0,903	-0.515	0.057	-9.026	0.000	REML	0

**Log-Av.Br.Len.$_{dist}$**	-0,198	0,042	-4,681	0,000	0.600	0.046	13.105	0.000	ML	0

Supplementary Table 3. Summary statistics of PGLSs (value, standard error, t-value and p-value) performed on femoral traits. BM is the abbreviation for Body Mass. Sample sizes for external shape analyses: n slow arboreal species= 16; n non-slow arboreal species=25; Sample sizes for mid-diaphyseal structure analyses: n slow arboreal species= 16; n non-slow arboreal species=26; Sample sizes for average diaphyseal structure analyses: n slow arboreal species= 15; n non-slow arboreal species=24; Sample sizes for proximal epiphyseal trabecular structure analyses: n slow arboreal species= 13; n non-slow arboreal species=23; Sample sizes for lateral condyle trabecular structure analyses: n slow arboreal species: 15; n non-slow arboreal species=23; Sample sizes for medial condyle trabecular structure analyses: n slow arboreal species: 14; n non-slow arboreal species=23.

Variable	Lifestyle.value	Lifestyle.Std.Err	Lifestyle.t.value	Lifestyle.p.value	BM.value	BM.Std.Err	BM.t.value	BM.p.value	method	λ
**3D GM PC1**	0.005	0.025	0.215	0.831	0.023	0.000	35718.144	0.000	ML	1
**3D GM PC2**	-0.034	0.013	-2.616	0.013	-0.021	0.011	-1.951	0.058	ML	0.997
**3D GM PC3**	0.016	0.008	1.868	0.069	-0.027	0.008	-3.273	0.002	REML	0.778
**3D GM PC4**	-0.003	0.009	-0.303	0.764	0.015	0.008	1.922	0.062	ML	0.975
**3D GM PC5**	-0.005	0.008	-0.662	0.512	0.013	0.007	1.734	0.091	REML	0
**3D GM PC6**	-0.019	0.006	-3.040	0.004	-0.014	0.006	-2.242	0.031	REML	0.514
**3D GM PC7**	-0.012	0.006	-2.073	0.045	0.002	0.006	0.318	0.752	ML	0.732
**3D GM PC8**	-0.008	0.004	-1.760	0.086	-0.006	0.004	-1.644	0.108	REML	0
**3D GM PC9**	0.003	0.006	0.426	0.672	-0.016	0.006	-2.727	0.010	REML	0.116
**3D GM PC10**	-0.006	0.004	-1.792	0.081	0.003	0.003	0.906	0.371	REML	0.072
**Log-ResC$_{50}$**	0.080	0.036	2.187	0.035	0.073	0.036	2.043	0.048	ML	0.575
**Log-ResC$_{Aver}$**	0.070	0.028	2.474	0.018	0.016	0.027	0.584	0.563	ML	0
**Log-Imax$_{50}$**	0.734	0.477	1.539	0.132	4.364	0.368	11.865	0.000	ML	1
**Log-Imax$_{Aver}$**	0.936	0.481	1.946	0.060	4.508	0.367	12.296	0.000	ML	1
**Log-Imin$_{50}$**	0.814	0.373	2.179	0.035	4.508	0.307	14.697	0.000	ML	0.998
**Log-Imin$_{Aver}$**	0.753	0.399	1.886	0.067	4.398	0.333	13.189	0.000	ML	0.997
**Log-CSA$_{50}$**	0.460	0.202	2.271	0.029	2.236	0.163	13.711	0.000	ML	0.999
**Log-CSA$_{Aver}$**	0.460	0.208	2.207	0.034	2.222	0.172	12.930	0.000	ML	0.998
**Log-CSS$_{50}$**	-0.090	0.159	-0.565	0.575	0.071	0.156	0.456	0.651	ML	0.74
**Log-CSS$_{Aver}$**	0.149	0.130	1.148	0.259	0.035	0.117	0.298	0.767	ML	0.973
**Log-DA$_{prox}$**	-0.180	0.068	-2.657	0.012	-0.067	0.070	-0.969	0.340	ML	0.498
**Log-Tb.Th.$_{prox}$**	0.024	0.074	0.328	0.745	0.553	0.075	7.368	0.000	ML	0.332
**Log-Conn.D.$_{prox}$**	-0.137	0.175	-0.780	0.441	-1.092	0.177	-6.178	0.000	REML	0.278
**BV.TV.$_{prox}$**	-0.091	0.020	-4.623	0.000	0.037	0.019	1.875	0.070	REML	0.159
**BS.TV.pr$_{ox}$**	-1.208	0.270	-4.477	0.000	-2.655	0.270	-9.843	0.000	REML	0.201
**DA$_{lat.con}$**	-0.138	0.042	-3.301	0.002	-0.071	0.046	-1.527	0.136	REML	0.691
**Log-Tb.Th.$_{lat.con}$**	0.185	0.055	3.354	0.002	0.573	0.061	9.456	0.000	ML	0.665
**Log-Conn.D.$_{lat.con}$**	-0.199	0.189	-1.056	0.298	-1.240	0.208	-5.960	0.000	REML	0.737

**Log-BV.TV.$_{lat.con}$**	0.037	0.040	0.937	0.355	0.147	0.041	3.551	0.001	REML	0.063
**Log-BS.TV.$_{lat.con}$**	-0.097	0.076	-1.278	0.210	-0.643	0.081	-7.955	0.000	ML	0.301
**Av.Br.Len.$_{lat.con}$**	0.027	0.015	1.712	0.096	0.188	0.016	11.413	0.000	ML	0.236
**DA$_{med.con}$**	-0.117	0.036	-3.256	0.003	-0.057	0.038	-1.479	0.148	ML	0.263
**Log-Tb.Th.$_{med.con}$**	0.049	0.079	0.618	0.541	0.497	0.085	5.865	0.000	ML	0.35
**Log-Conn.D.$_{med.con}$**	-0.016	0.165	-0.094	0.926	-1.177	0.179	-6.588	0.000	REML	0.421
**Log-BV.TV.$_{med.con}$**	-0.115	0.069	-1.670	0.104	0.130	0.073	1.794	0.082	REML	0.127
**Log-BS.TV.$_{med.con}$**	-0.118	0.125	-0.939	0.354	-0.727	0.126	-5.761	0.000	ML	1
**Log-Av.Br.Len.$_{med.con}$**	0.062	0.063	0.998	0.325	0.607	0.067	9.108	0.000	ML	0.247

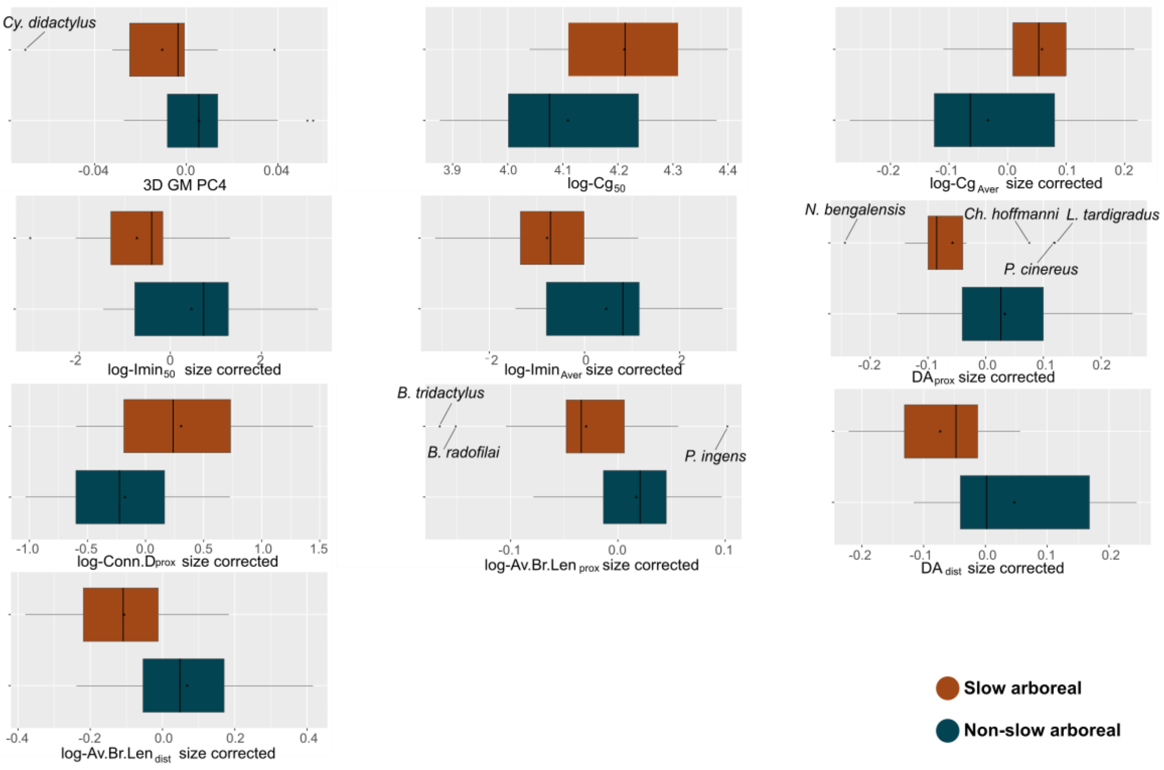

Supplementary Fig. 2. Boxplots with the distribution of mean taxa results for humeral traits that yielded a significant relationship with slow arboreal ecology through PGLSs and ANCOVAs. Structural traits showing a significant correlation with body mass too are shown with size-corrected values. Each box starts with 1st quartile, ends with the 3rd quartile and contains the mean (shown with a black triangle) and the median (shown with a vertical line). Whiskers indicate minimum and maximum values. Slow and non-slow arboreal species sample sizes for each humeral anatomical level and/or sub-region are detailed in the caption of Supplementary Tables 2-3.

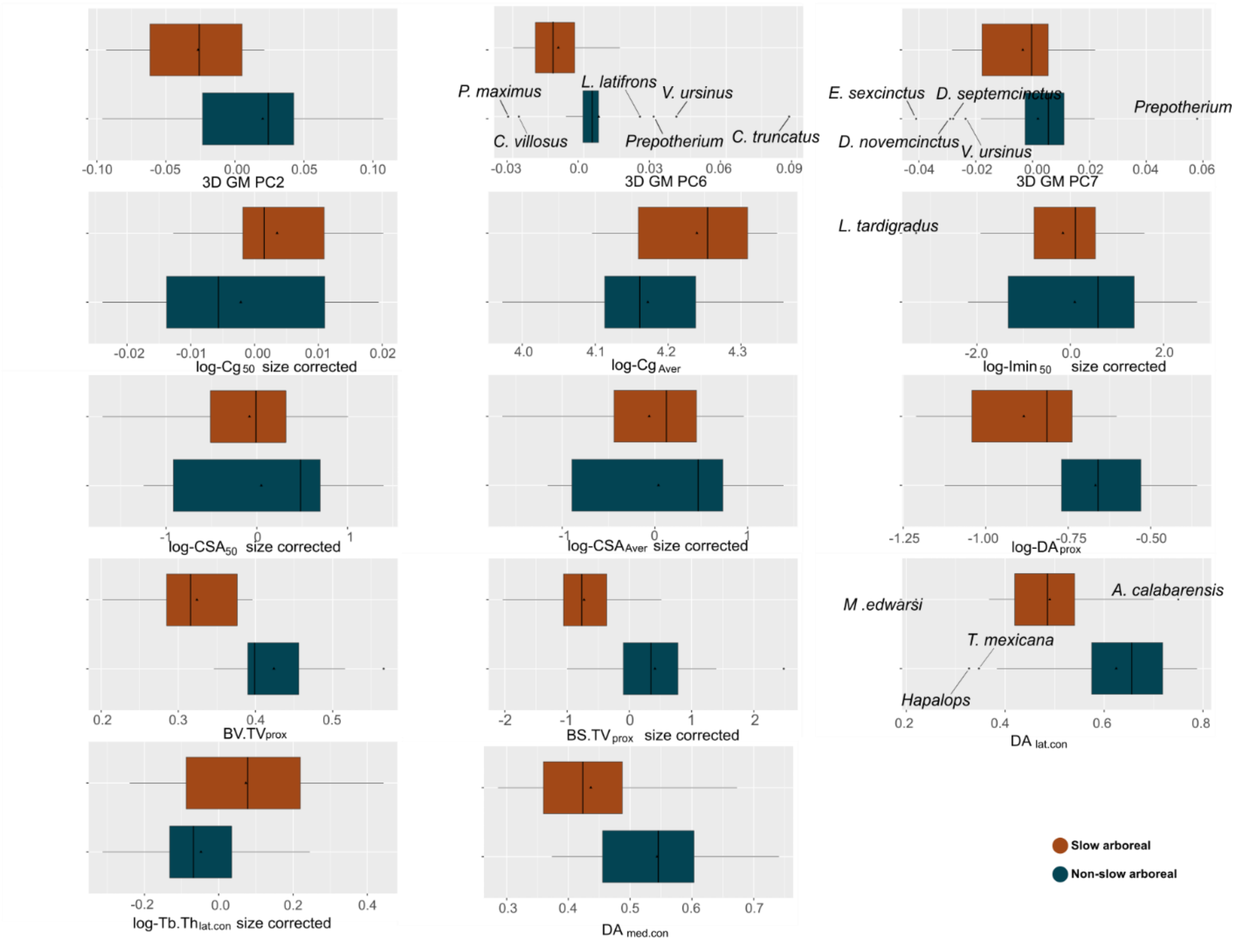

Supplementary Fig. 3. Boxplots with the distribution of mean taxa results for femoral traits that yielded a significant relationship with slow arboreal ecology through PGLSs and ANCOVAs. Structural traits showing a significant correlation with body mass too are shown with size-corrected values. Each box starts with 1st quartile, ends with the 3rd quartile and contains the mean (shown with a black triangle) and the median (shown with a vertical line). Whiskers indicate minimum and maximum values. Slow and non-slow arboreal species sample sizes for each femoral anatomical level and/or sub-region are detailed in the caption of Supplementary Tables 2-3.

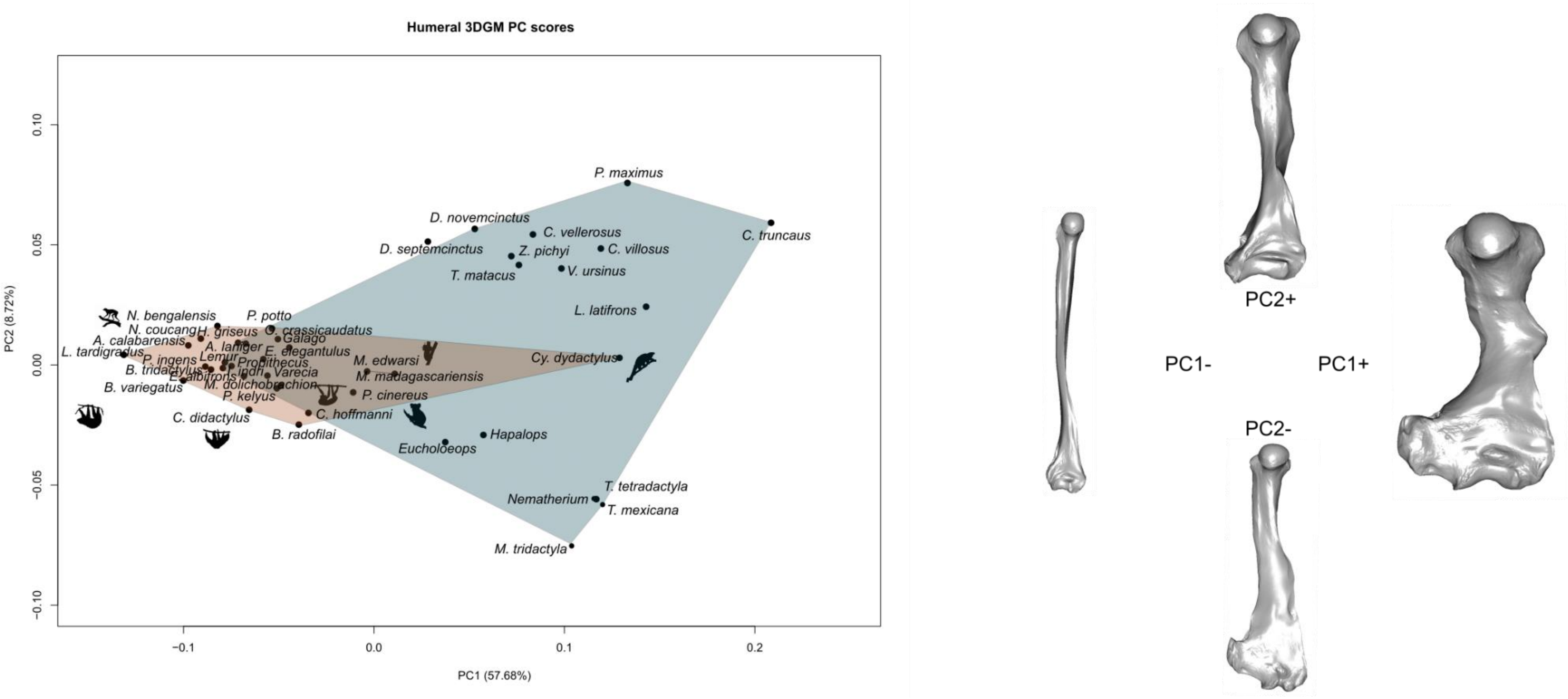

Supplementary Fig. 4. Left panel: Humeral shape variability, highlighted through a 3DGM PC1-PC2 biplot. Slow arboreal mammals are shown in red (together with silhouettes for the seven main clades) while non slow arboreal mammals are shown in blue. Right panel: the main shape variability captured by PC1 and PC2 is shown through maximum/minimum PC1 and PC2 scores warped on the mesh of *Eucholoeops* sp. FMNH P13280 (additionally inflated in the diaphyseal region and smoothed to delete surface micro-cracks, to optimise the warping process).

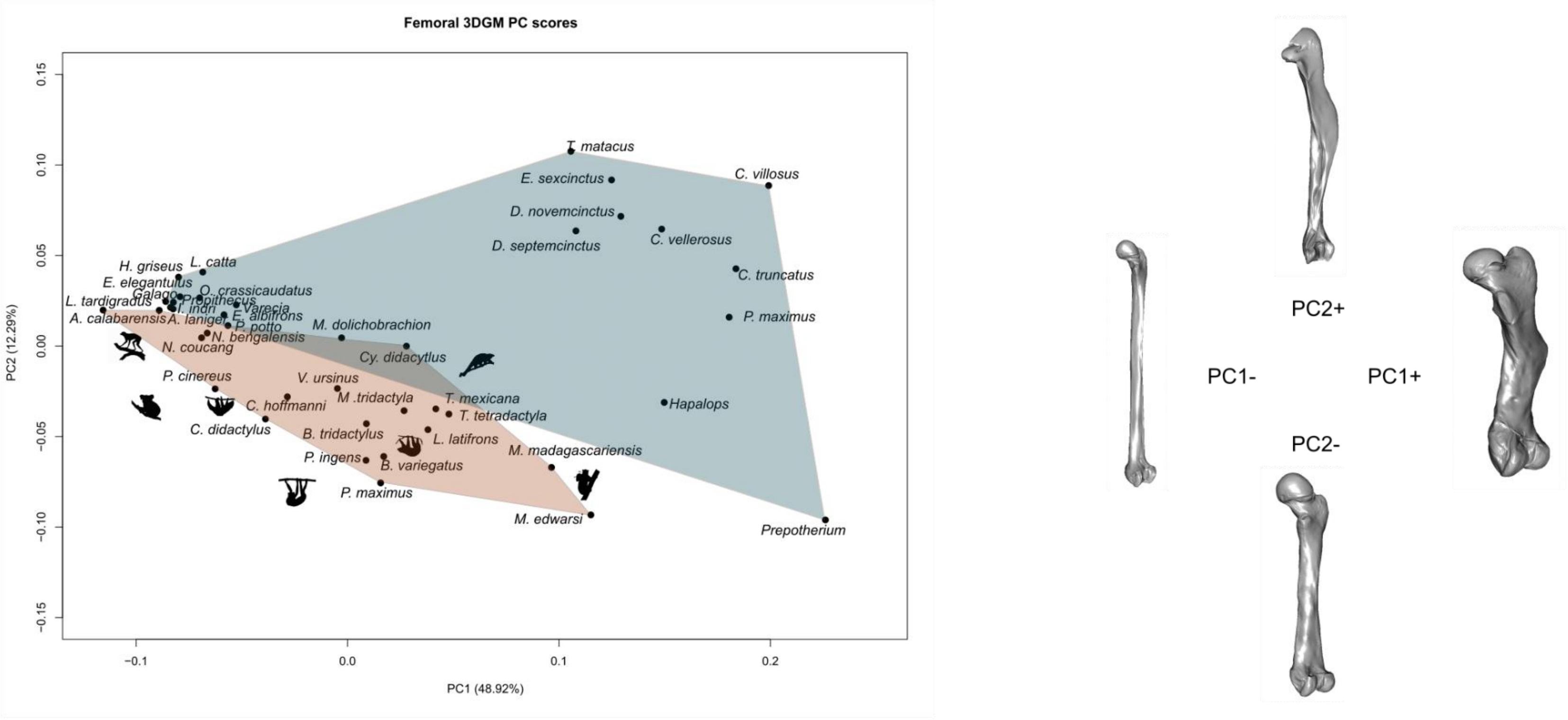

Supplementary Fig. 5. Left panel: Femoral shape variability, highlighted through a 3DGM PC1-PC2 biplot. Slow arboreal mammals are shown in red (together with silhouettes for the seven main clades) while non slow arboreal mammals are shown in blue. Right panel: the main shape variability captured by PC1 and PC2 is shown through maximum/minimum PC1 and PC2 scores warped on the mesh of *Bradypus* sp. ZMB Mam-33806 (additionally inflated in the diaphyseal region to optimise the warping process).

Supplementary Table 4. Summary statistics for each of the Principal Component deriving from PCA on humeral shape landmark coordinates

	Standard deviation	Proportion of Variance	Cumulative Proportion
**PC1**	0.086390117	0.57686	0.57686
**PC2**	0.033576615	0.08714	0.664
**PC3**	0.025596339	0.05064	0.71464
**PC4**	0.025047094	0.04849	0.76313
**PC5**	0.022830685	0.04029	0.80342
**PC6**	0.018336258	0.02599	0.8294
**PC7**	0.017412081	0.02343	0.85284
**PC8**	0.014110671	0.01539	0.86823
**PC9**	0.013150626	0.01337	0.88159
**PC10**	0.012941417	0.01295	0.89454
**PC11**	0.011479979	0.01019	0.90473
**PC12**	0.010658432	0.00878	0.91351
**PC13**	0.010469838	0.00847	0.92198
**PC14**	0.008943498	0.00618	0.92816
**PC15**	0.008178377	0.00517	0.93333
**PC16**	0.00791441	0.00484	0.93817
**PC17**	0.007292765	0.00411	0.94228
**PC18**	0.006866177	0.00364	0.94593
**PC19**	0.006584561	0.00335	0.94928
**PC20**	0.006510077	0.00328	0.95255
**PC21**	0.006373732	0.00314	0.95569
**PC22**	0.005986795	0.00277	0.95847
**PC23**	0.005913278	0.0027	0.96117
**PC24**	0.005592926	0.00242	0.96359
**PC25**	0.005508396	0.00235	0.96593
**PC26**	0.005446737	0.00229	0.96822
**PC27**	0.005141569	0.00204	0.97027
**PC28**	0.004885436	0.00184	0.97211
**PC29**	0.004745762	0.00174	0.97385
**PC30**	0.004556859	0.0016	0.97546
**PC31**	0.004372742	0.00148	0.97694
**PC32**	0.004236197	0.00139	0.97832
**PC33**	0.004154547	0.00133	0.97966
**PC34**	0.004015473	0.00125	0.9809
**PC35**	0.003858012	0.00115	0.98205
**PC36**	0.003720743	0.00107	0.98312
**PC37**	0.003613121	0.00101	0.98413
**PC38**	0.003476471	0.00093	0.98507
**PC39**	0.003372416	0.00088	0.98595
**PC40**	0.003315685	0.00085	0.9868

**PC41**	0.00309111	0.00074	0.98753
**PC42**	0.003019842	0.0007	0.98824
**PC43**	0.002881965	0.00064	0.98888
**PC44**	0.002814742	0.00061	0.98949
**PC45**	0.002733165	0.00058	0.99007
**PC46**	0.002689559	0.00056	0.99063
**PC47**	0.002589704	0.00052	0.99115
**PC48**	0.002559398	0.00051	0.99165
**PC49**	0.002481088	0.00048	0.99213
**PC50**	0.002461728	0.00047	0.9926
**PC51**	0.002373761	0.00044	0.99303
**PC52**	0.002323164	0.00042	0.99345
**PC53**	0.002265849	0.0004	0.99385
**PC54**	0.002186795	0.00037	0.99422
**PC55**	0.002131233	0.00035	0.99457
**PC56**	0.002072322	0.00033	0.9949
**PC57**	0.00200184	0.00031	0.99521
**PC58**	0.001922563	0.00029	0.9955
**PC59**	0.001873157	0.00027	0.99577
**PC60**	0.001852904	0.00027	0.99603
**PC61**	0.001807657	0.00025	0.99629
**PC62**	0.001765282	0.00024	0.99653
**PC63**	0.001735454	0.00023	0.99676
**PC64**	0.001700403	0.00022	0.99698
**PC65**	0.001653217	0.00021	0.99719
**PC66**	0.001612246	0.0002	0.99739
**PC67**	0.001585923	0.00019	0.99759
**PC68**	0.001530825	0.00018	0.99777
**PC69**	0.001476223	0.00017	0.99794
**PC70**	0.001451354	0.00016	0.9981
**PC71**	0.001403012	0.00015	0.99825
**PC72**	0.001361403	0.00014	0.9984
**PC73**	0.001304945	0.00013	0.99853
**PC74**	0.00128246	0.00013	0.99866
**PC75**	0.001226926	0.00012	0.99877
**PC76**	0.001210376	0.00011	0.99889
**PC77**	0.001180231	0.00011	0.99899
**PC78**	0.001155421	0.0001	0.9991
**PC79**	0.001129408	0.0001	0.99919
**PC80**	0.001114161	0.0001	0.99929
**PC81**	0.001071673	0.00009	0.99938
**PC82**	0.001043801	0.00008	0.99946
**PC83**	0.000991104	0.00008	0.99954
**PC84**	0.000978648	0.00007	0.99961

**PC85**	0.000917754	0.00007	0.99968
**PC86**	0.000914688	0.00006	0.99974
**PC87**	0.000882844	0.00006	0.9998
**PC88**	0.000862166	0.00006	0.99986
**PC89**	0.000808498	0.00005	0.99991
**PC90**	0.000766047	0.00005	0.99996
**PC91**	0.000746121	0.00004	1
**V92**	4.83723E-17	0	1

Supplementary Table 5. Summary statistics for each of the Principal Component deriving from PCA on femoral shape landmark coordinates

	Standard deviation	Proportion of Variance	Cumulative Proportion
**PC1**	0.087257341	0.48915	0.48915
**PC2**	0.04373946	0.12291	0.61206
**PC3**	0.033371725	0.07155	0.68361
**PC4**	0.026915934	0.04654	0.73016
**PC5**	0.022119917	0.03143	0.76159
**PC6**	0.018720619	0.02252	0.78411
**PC7**	0.017405328	0.01946	0.80357
**PC8**	0.016866013	0.01828	0.82184
**PC9**	0.016376882	0.01723	0.83907
**PC10**	0.014913138	0.01429	0.85336
**PC11**	0.013773994	0.01219	0.86555
**PC12**	0.012816844	0.01055	0.8761
**PC13**	0.012623898	0.01024	0.88634
**PC14**	0.011537569	0.00855	0.8949
**PC15**	0.010984713	0.00775	0.90265
**PC16**	0.010772123	0.00745	0.9101
**PC17**	0.010487255	0.00707	0.91717
**PC18**	0.009712688	0.00606	0.92323
**PC19**	0.009318869	0.00558	0.92881
**PC20**	0.008630406	0.00479	0.93359
**PC21**	0.008305151	0.00443	0.93802
**PC22**	0.008212806	0.00433	0.94236
**PC23**	0.007496495	0.00361	0.94597
**PC24**	0.00727566	0.0034	0.94937
**PC25**	0.007067396	0.00321	0.95258
**PC26**	0.006923676	0.00308	0.95566
**PC27**	0.006859189	0.00302	0.95868
**PC28**	0.006684497	0.00287	0.96155
**PC29**	0.006141374	0.00242	0.96397
**PC30**	0.0059277	0.00226	0.96623
**PC31**	0.005851318	0.0022	0.96843
**PC32**	0.005768891	0.00214	0.97057
**PC33**	0.005419212	0.00189	0.97246
**PC34**	0.005248069	0.00177	0.97423
**PC35**	0.004988213	0.0016	0.97582
**PC36**	0.004621877	0.00137	0.9772
**PC37**	0.004460855	0.00128	0.97847
**PC38**	0.004359045	0.00122	0.9797
**PC39**	0.004237487	0.00115	0.98085

**PC40**	0.004053661	0.00106	0.98191
**PC41**	0.003987157	0.00102	0.98293
**PC42**	0.003795867	0.00093	0.98385
**PC43**	0.003651958	0.00086	0.98471
**PC44**	0.003617738	0.00084	0.98555
**PC45**	0.003553155	0.00081	0.98636
**PC46**	0.00336606	0.00073	0.98709
**PC47**	0.00329498	0.0007	0.98779
**PC48**	0.003254455	0.00068	0.98847
**PC49**	0.00311682	0.00062	0.98909
**PC50**	0.003092249	0.00061	0.98971
**PC51**	0.003040483	0.00059	0.9903
**PC52**	0.002945774	0.00056	0.99086
**PC53**	0.002840138	0.00052	0.99137
**PC54**	0.002720423	0.00048	0.99185
**PC55**	0.002698611	0.00047	0.99232
**PC56**	0.002556764	0.00042	0.99274
**PC57**	0.002489704	0.0004	0.99314
**PC58**	0.002435787	0.00038	0.99352
**PC59**	0.002362807	0.00036	0.99388
**PC60**	0.002350858	0.00036	0.99423
**PC61**	0.002332169	0.00035	0.99458
**PC62**	0.002268237	0.00033	0.99491
**PC63**	0.002188939	0.00031	0.99522
**PC64**	0.002158813	0.0003	0.99552
**PC65**	0.002119277	0.00029	0.99581
**PC66**	0.00205034	0.00027	0.99608
**PC67**	0.0020044	0.00026	0.99634
**PC68**	0.001990129	0.00025	0.99659
**PC69**	0.001927432	0.00024	0.99683
**PC70**	0.001872881	0.00023	0.99705
**PC71**	0.0018269	0.00021	0.99727
**PC72**	0.001770852	0.0002	0.99747
**PC73**	0.001757868	0.0002	0.99767
**PC74**	0.001720595	0.00019	0.99786
**PC75**	0.001688665	0.00018	0.99804
**PC76**	0.001629327	0.00017	0.99821
**PC77**	0.001580868	0.00016	0.99837
**PC78**	0.001568801	0.00016	0.99853
**PC79**	0.001546129	0.00015	0.99868
**PC80**	0.001539381	0.00015	0.99884
**PC81**	0.001494321	0.00014	0.99898
**PC82**	0.001430808	0.00013	0.99911
**PC83**	0.001400231	0.00013	0.99924

**PC84**	0.001389099	0.00012	0.99936
**PC85**	0.001360814	0.00012	0.99948
**PC86**	0.001315721	0.00011	0.99959
**PC87**	0.001251368	0.0001	0.99969
**PC88**	0.001205606	0.00009	0.99979
**PC89**	0.001127584	0.00008	0.99987
**PC90**	0.001077373	0.00007	0.99994
**PC91**	0.000951747	0.00006	1
**V92**	4.61186E-17	0	1

Slow arboreal mammals: Scheme A						
Humerus						
	C1	p-C1	C2	p-C2	C3	p-C3
3D GM	0.664	0.128	***0.033***	***<0.001***	0.399	0.105
CSP_{50}	***0.27***	***0.029***	***0.508***	***0.002***	0.128	0.060
CSP_{Aver}	***0.34***	***0.005***	***0.63***	***<0.001***	***0.166***	***0.006***
$Trab_{prox}$	***0.25***	***0.025***	***0.537***	***0.001***	***0.146***	***0.020***
$Trab_{dist}$	***0.34***	***0.012***	***0.708***	***<0.001***	***0.178***	***0.012***
Femur						
	C1	p-C1	C2	p-C2	C3	p-C3
3D GM	0.229	0.060	***0.014***	***0.048***	0.112	0.108
CSP_{50}	0.268	0.056	***0.567***	***0.004***	0.136	0.079
CSP_{Aver}	0.197	0.140	***0.305***	***0.024***	0.096	0.229
$Trab_{prox}$	***0.294***	***0.001***	***0.617***	***<0.001***	***0.137***	***0.002***
$Trab_{lat,con}$	***0.255***	***0.032***	***0.507***	***<0.001***	0.124	0.077
$Trab_{med,con}$	0.146	0.331	0.016	0.183	0.128	0.323

Slow arboreal mammals excluding (*Arctocebus* + *Perodicticus*): Scheme B						
Humerus						
	C1	p-C1	C2	p-C2	C3	p-C3
3D GM	0.664	0.120	***0.033***	***<0.001***	0.399	0.102
CSP_{50}	***0.31***	***0.012***	***0.59***	***0.001***	***0.143***	***0.024***
CSP_{Aver}	***0.348***	***0.003***	***0.669***	***<0.001***	***0.167***	***0.006***
$Trab_{prox}$	0.234	0.074	***0.511***	***0.008***	0.132	0.053
$Trab_{dist}$	***0.329***	***0.026***	***0.677***	***0.001***	***0.169***	***0.039***
Femur						
	C1	p-C1	C2	p-C2	C3	p-C3
3D GM	***0.272***	***0.027***	***0.016***	***0.018***	0.130	0.058
CSP_{50}	***0.280***	***0.045***	***0.584***	***0.006***	0.130	0.137
CSP_{Aver}	0.196	0.198	0.298	0.057	0.088	0.395
$Trab_{prox}$	***0.3374***	***0.001***	***0.7387***	***<0.001***	***0.162***	***0.003***
$Trab_{lat,con}$	***0.3406***	***0.004***	***0.6764***	***<0.001***	***0.1657***	***0.012***
$Trab_{med,con}$	0.146	0.347	0.0167	0.160	0.128	0.341

Slow arboreal mammals excluding (*Nycticebus* + *Loris*): Scheme C						
Humerus						
	C1	p-C1	C2	p-C2	C3	p-C3
3D GM	0.664	0.120	***0.033***	***<0.001***	0.399	0.094
CSP_{50}	***0.279***	***0.031***	***0.511***	***0.003***	0.131	0.060
CSP_{Aver}	***0.351***	***0.005***	***0.628***	***0.001***	***0.168***	***0.012***
$Trab_{prox}$	0.224	0.088	***0.507***	***0.007***	0.128	0.064
$Trab_{dist}$	***0.362***	***0.013***	***0.783***	***<0.001***	***0.194***	***0.010***
Femur						
	C1	p-C1	C2	p-C2	C3	p-C3
3D GM	0.240	0.059	***0.014***	***0.048***	0.109	0.168
CSP_{50}	***0.318***	***0.014***	***0.669***	***0.002***	***0.158***	***0.024***
CSP_{Aver}	0.222	0.087	***0.332***	***0.025***	0.103	0.196
$Trab_{prox}$	***0.285***	***<0.001***	***0.505***	***<0.001***	***0.120***	***0.011***
$Trab_{lat,con}$	***0.315***	***0.014***	***0.617***	***<0.001***	***0.1473***	***0.027***
$Trab_{med,con}$	0.1468	0.344	0.0167	0.172	0.128	0.338

Supplementary Table 6. C1-C3 values and relative p-values for each convergence analysis. Significant C-indices and p-values are highlighted in bold. Slow and non-slow arboreal species sample sizes for each bone, anatomical level and/or sub-region are detailed in the caption of Supplementary Tables 2-3.

Supplementary Table 7. Summary statistics for each of the Principal Component deriving from PCA on humeral traits previously found to significantly discriminate slow arboreal from non-slow arboreal mammals

	Standard deviation	Proportion of Variance	Cumulative Proportion
**PC1**	2.163388	0.46802	0.46802
**PC2**	1.442466	0.20807	0.6761
**PC3**	1.046555	0.10953	0.78562
**PC4**	0.844697	0.07135	0.85697
**PC5**	0.747684	0.0559	0.91288
**PC6**	0.689062	0.04748	0.96036
**PC7**	0.467056	0.02181	0.98217
**PC8**	0.323525	0.01047	0.99264
**PC9**	0.259401	0.00673	0.99937
**PC10**	0.079472	0.00063	1

Supplementary Table 8. Summary statistics for each of the Principal Component deriving from PCA on femoral traits previously found to significantly discriminate slow arboreal from non-slow arboreal mammals

	Standard deviation	Proportion of Variance	Cumulative Proportion
**PC1**	2.177035	0.33853	0.33853
**PC2**	1.843526	0.24276	0.58129
**PC3**	1.252744	0.1121	0.69339
**PC4**	1.018938	0.07416	0.76755
**PC5**	0.979285	0.0685	0.83605
**PC6**	0.810787	0.04696	0.883
**PC7**	0.75814	0.04106	0.92406
**PC8**	0.62333	0.02775	0.95181
**PC9**	0.549699	0.02158	0.9734
**PC10**	0.474244	0.01606	0.98946
**PC11**	0.349896	0.00874	0.9982
**PC12**	0.141284	0.00143	0.99963
**PC13**	0.066166	0.00031	0.99994
**PC14**	0.028167	0.00006	1

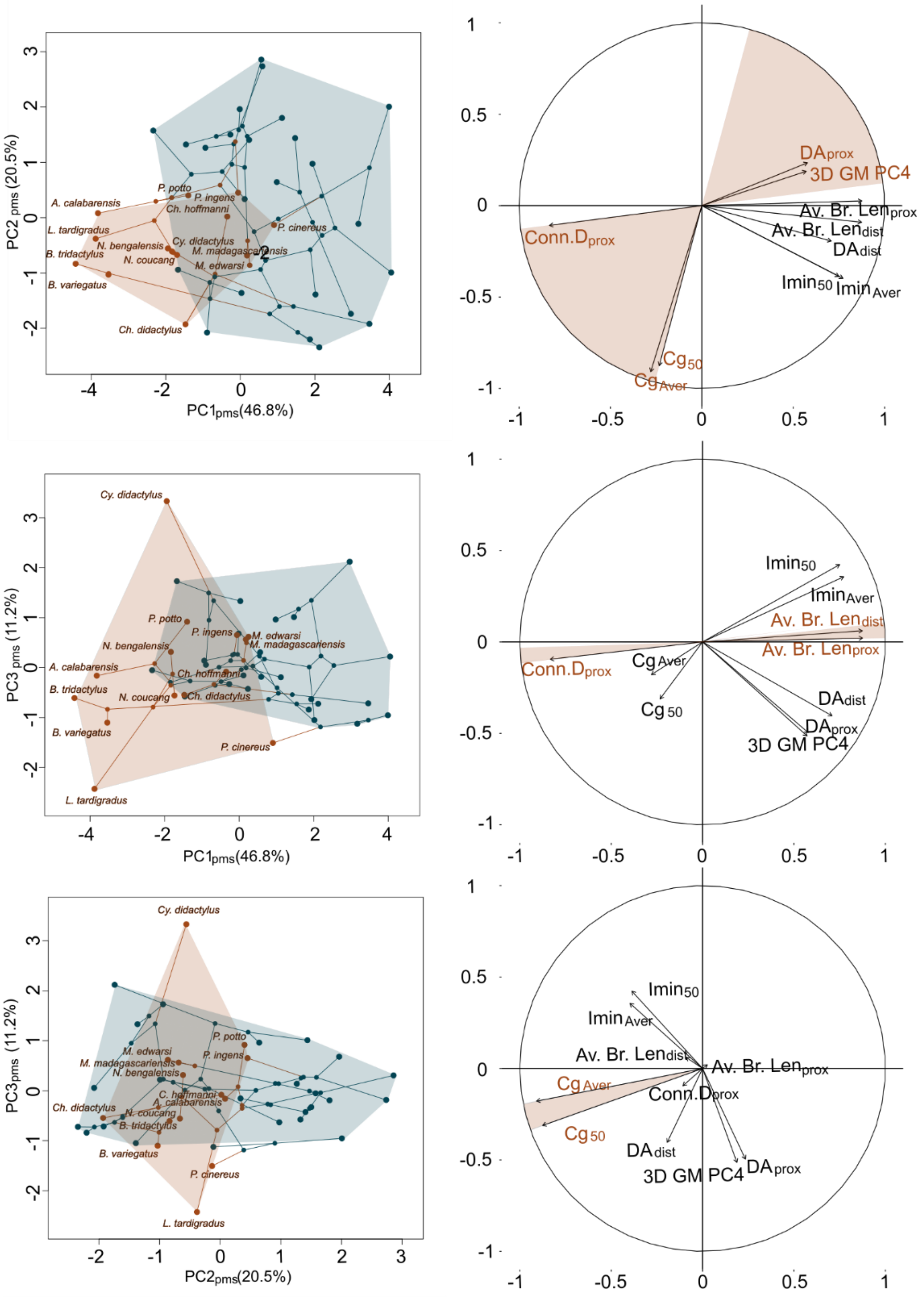

Supplementary Fig. 6. Left panel: 2D phylomorphospaces based on $PC1_{pms}$-$PC2_{pms}$, $PC1_{pms}$-$PC3_{pms}$ and $PC2_{pms}$-$PC3_{pms}$ biplots, with PC_{pms} deriving from Principal Component Analyses on humeral external shape and inner structural traits that significantly set apart slow arboreal (shown in red) from non-slow arboreal mammals (shown in blue). Right panel: for each 2D phylomorphospace the related variable loadings plot is shown, with variables mostly contributing to the similarity of slow arboreal mammals that are highlighted in orange.

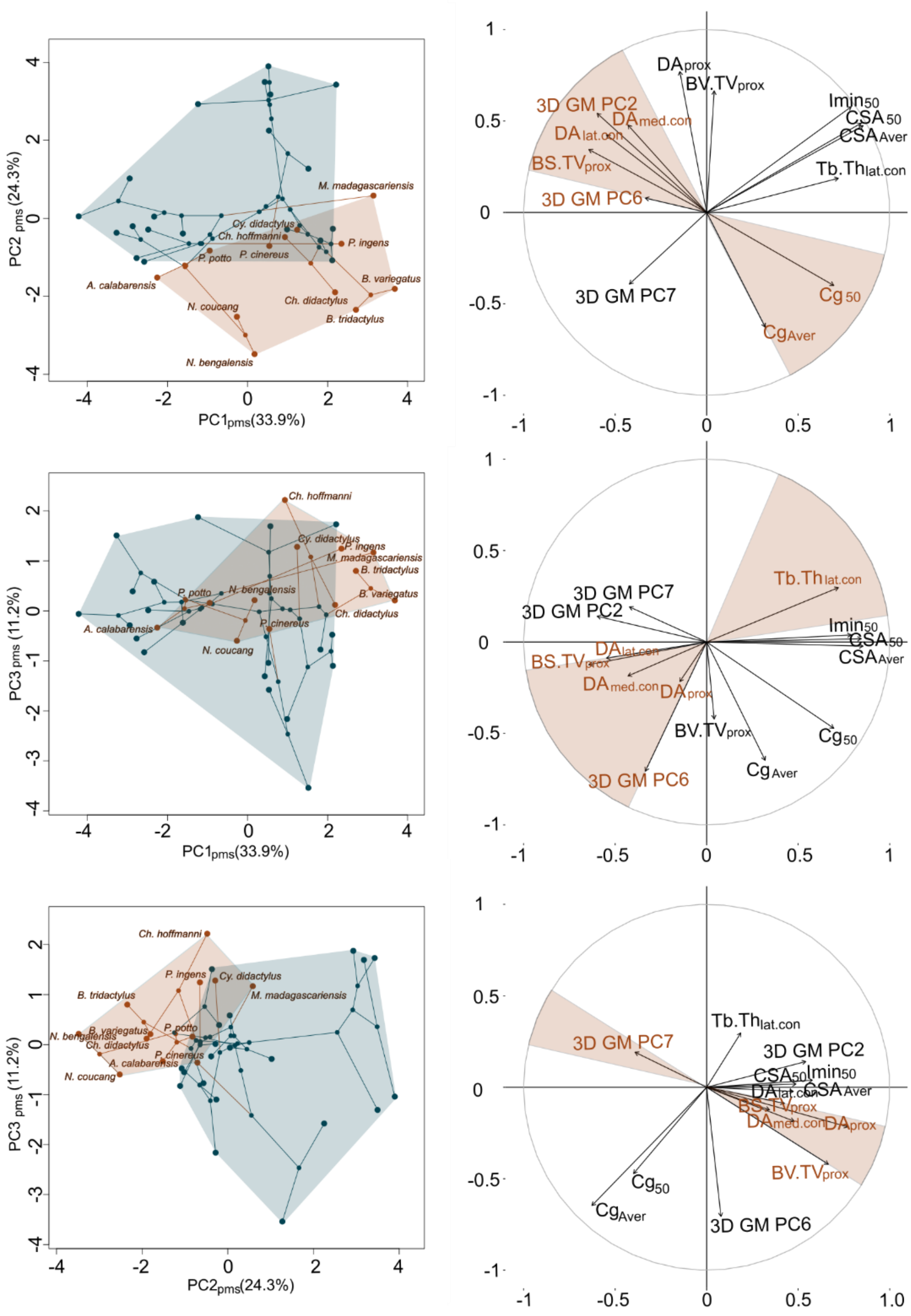

Supplementary Fig. 7. Left panel: 2D phylomorphospaces based on $PC1_{pms}$-$PC2_{pms}$, $PC1_{pms}$-$PC3_{pms}$ and $PC2_{pms}$-$PC3_{pms}$ biplots, with PC_{pms} deriving from Principal Component Analyses on femoral external shape and inner structural traits that significantly set apart slow arboreal (shown in red) from non-slow arboreal mammals (shown in blue). Right panel: for each 2D phylomorphospace the related variable loadings plot is shown, with variables mostly contributing to the similarity of slow arboreal mammals that are highlighted in orang

Supplementary Notes

1. Collections visited and abbreviation in specimen catalogue number

- Museum für Naturkunde, Berlin, Germany (ZMB Mam)
- Staatliches Museum für Naturkunde, Stuttgart, Germany (SMNS)
- Zoologisches Forschungsmuseum Alexander Koenig, Bonn, Germany (ZFMK Mam)
- Zoologische Staatssammlung, Munich, Germany (ZSM)
- Naturhistorisches Museum, Wien, Austria (NMW)
- Muséum national d'Histoire naturelle, Paris, France (MNHN)
- American Museum of Natural History, New York, NY, USA (AMNH)
- Field Museum of Natural History, Chicago, IL, USA (FMNH)
- Yale Peabody Museum of Natural History, New Haven, CT, USA (YPM PU)
- Division of Fossil Primates, Duke Lemur Center, Durham, NC, USA (DCP)

2. Taxonomic assignments

Extant taxa

One humerus and one femur belonging to an individual not classified at the species level (*Bradypus* sp ZMB Mam 33806) from the sample of Alfieri et al.[1] were not included in this work (the femur was only used to drive 3D GM data extraction but not further analysed, see below). Specimens not catalogued at the species level often prevent to completely inform dataframes with phylogenies and cause the exclusion from phylogenetically informed comparative analyses (unless the phylogeny is accordingly adjusted; see below). While the exclusion of these specimens of *Bradypus* has minor effects on the representation of the genus, following the same procedure for all the taxa would have resulted in widely decreased sample size for *Galago*, *Varecia* and *Propithecus*. To avoid underrepresenting these genera, we pooled all their specimens in *Galago* sp., *Varecia* sp. and *Propithecus* sp., respectively. This was possible since, for these three genera, specimens do not show wide variation in body mass proxy (Supplementary Data 1-2) and, in this work, species of the same genus always belong to the same ecological class. Bones classified as *Lemur* sp. (from ZMB Mam 83963 and ZMB Mam 83964), were assigned to *Lemur catta*, it being the only extant species within the genus.

Extinct taxa

We considered fossil specimens of extinct sloths at the genus taxonomic level (Supplementary Data 1-2). It is justified by the absence of relevant inferred size and ecological variation within the same genus and it is motivated by the difficulty to phylogenetically inform morphological data taken from species level-catalogued fossil xenarthran bones. Indeed, phylogenetic reconstructions of fossil sloths, both concerning evolutionary relationships and divergence times, generally only consider the genus level[2–4]. As for specimens of subfossil lemurs, instead, we were forced to classify them at the species level due to the wide variation in bone size within some genera (Supplementary Data 1-2). We based taxonomic assignments on Godfrey et al.[5,6] and Jungers et al.[7]'s work. Body mass data inferred by Godfrey et al.[5,6] were used by Jungers et al. to compute mass-adjusted humeral and femoral lengths for subfossil lemur species[7]. Using data from Godfrey et al. and Jungers et al.[5–7] and the Jungers et al.'s formulae of mass-adjusted lenghts[7], we extracted a humeral and femoral mean absolute maximum length for each species of subfossil lemur. Measured humeral and femoral lengths of complete bones from our sample were then compared to reference mean absolute length values, assigning each specimen to the species with the closest mean absolute length. Incomplete specimens (i.e. isolated epiphyses) were assigned to the species to which belongs the complete subfossil lemur specimen with the closest epiphyseal metric measurements (see main text for details on these epiphyseal measures). One humerus of *Palaeopropithecus* (DPC 11861) is evidently shorter than the other humeri of the genus and its length is widely smaller than the reconstructed mean absolute lengths of all the species of *Palaeopropithecus*. We assigned this humerus to *P. kelyus*. This species was discovered later than Jungers et al.'s work[7] by Gommery et al.[8], hence we did not have comparative data of bone lengths. However, the smaller size compared to other two species of *Palaeopropithecus* (*P. maximus* and *P. ingens*) is one of the main characteristics of *P. kelyus* ('kelyus' comes from 'kely', i.e. 'small' in Malagasy)[8,9]. Moreover, the humerus of *Palaeopropithecus* DPC 11861 comes from Anjohibe (Northern Madagascar) and the species *P. kelyus* was originally discovered and defined based on specimens found in several sites in the North-West of Madagascar, including a basin at Anjohibe[8]. The epiphyseal metric measurements of the incomplete humerus (i.e. proximal epiphysis) of *Palaeopropithecus* DPC 17342 fall between those of *P. kelyus* and those of *P. ingens*. Again, we exploited palaeontological evidence to assign this specimen to a species. *Palaeopropithecus* DPC 17342 comes from Ankilitelo Cave (South-West of Madagascar), an area in which *P. keylus* has not been yet retrieved (although recent findings extended south the geographic distribution of *P. kelyus*[10], it still does not reach the area of Ankilitelo Cave, to the best of our knowledge). Thus, we assigned *Palaeopropithecus* DPC 17342 to *P. ingens*. Specimens catalogued as *Babakotia* sp. were assigned to *Babakotia radofilai*, only known species for the genus. Results of the taxonomic assignments for and data justifying them are detailed as following:

Species	Estimated body mass (kg)[a]	Mass-adjusted humeral length[a]	Mass-adjusted femoral length[a]	Mean max humeral length (mm)[b]	Mean max femoral length (mm)[b]
Mesopropithecus globiceps	9.4	57.5	63.1	121.35	133.17
Mesopropithecus pithecoides	9.7	61.7	67.6	131.59	144.17
Mesopropithecus dolicobrachion	10.6	66.1	63.1	145.20	138.61
Babakotia radofilai	16.2	70.8	61.7	179.15	156.12
Palaeopropithecus ingens	45.4	79.8	53.7	284.68	191.57
Palaeopropithecus maximus	52.3	79.7	53	298.05	198.20
Megaladapis madagascariensis	38	55	49	184.91	164.74
Megaladapis grandidieri	63	53.7	46.8	213.68	186.22
Megaladapis edwarsi	75.4	57.5	52.5	242.92	221.79

Data on Malagasy subfossil lemurs taken from literature and used as reference for taxonomic assignment for specimens studied in this work

[a] data taken from Godfrey et al.[5,6] and Jungers et al.[7]

[b] data extracted entering data from Godfrey et al.[5,6] and Jungers et al.[7] in Jungers et al.[7]'s formulae:

- $Mass\ adjusted\ humeral\ length = \frac{Mean\ \max humeral\ length\ (mm)}{\sqrt[3]{Body\ mass\ (kg)}}$
- $Mass\ adjusted\ femoral\ length = \frac{Mean\ \max femoral\ length\ (mm)}{\sqrt[3]{Body\ mass\ (kg)}}$

Complete bones					
**Humeri (using comparison with data above)**			**Femora (using comparison with data above)**		
**Specimens**	**Length (mm)**	**Assigned to**	**Specimens**	**Length (mm)**	**Assigned to**
Babakotia sp DPC 11824	167.111	*Babakotia radofilai*	*Megaladapis* sp MNHM MAD-1567	156.55	*Megaladapis madagascariensis*
Megaladapis sp MNHN MAD-1562	184.541	*Megaladapis madagascariensis*	*Megaladapis* sp MNHM MAD-7403	210.46	*Megaladapis edwarsi*
Megaladapis sp MNHN MAD-7777	257.562	*Megaladapis edwarsi*	*Megaladapis* sp MNHM MAD-7405	215.89	*Megaladapis edwarsi*
Megaladapis sp MNHN MAD-7778	244.003	*Megaladapis edwarsi*	*Palaeopropithecus* sp DCP 17342	199.645	*Palaeopropithecus maximus*
Palaeopropithecus sp DPC 11861	178.386	*Palaeopropithecus kelyus*	*Palaeopropithecus* sp DCP UA5469	176.92	*Palaeopropithecus ingens*
Palaeopropithecus sp DPC UA5465	273.28	*Palaeopropithecus ingens*	*Palaeopropithecus* sp MNHM MAD-8551	183.335	*Palaeopropithecus ingens*
Palaeopropithecus sp DPC UA5474	274.16	*Palaeopropithecus ingens*	*Palaeopropithecus* sp MNHM MAD-8795	181.75	*Palaeopropithecus ingens*

Palaeopropithecus sp MNHN MAD-8605	274.606	*Palaeopropithecus ingens*	*Palaeopropithecus* sp MNHM MAD-8808	194.94	*Palaeopropithecus ingens*

Humeral (left) and femoral (right) lengths, directly measured on the complete Malagasy subfossil lemur specimens studied in this work were compared to the mean humeral and femoral length values of the subfossil lemur species, extracted as detailed above. Specimens were assigned to the species with the closest estimated length.

**Non-complete bones**			
**Humeri (using comparison with epiphyseal measurements)**		**Femora (using comparison with epiphyseal measurements)**	
**Specimens**	**Assigned to**	**Specimens**	**Assigned to**
Babakotia sp DPC 11825_onlyDist	*Babakotia radofilai*	*Megaladapis* sp DPC 17225_onlyDist	*Megaladapis edwarsi*
Megaladapis sp MNHN MAD-1561_onlyProx	*Megaladapis madagascariensis*	*Megaladapis* sp MNHN MAD-1564_onlyDist	*Megaladapis madagascariensis*
Megaladapis sp MNHN MAD-7374_onlyDist	*Megaladapis madagascariensis*	*Megaladapis* sp MNHN MAD-1564_onlyProx	*Megaladapis madagascariensis*
Palaeopropithecus sp DPC 17342_onlyProx	*Palaeopropithecus ingens*	*Megaladapis* sp MNHN MAD-7429_onlyProx	*Megaladapis edwarsi*
Palaeopropithecus sp MNHN MAD-8594_onlyDist	*Palaeopropithecus ingens*	*Palaeopropithecus* sp DPC 17150_onlyDist	*Palaeopropithecus ingens*
		Palaeopropithecus sp MNHN MAD-1584_onlyProx	*Palaeopropithecus ingens*

Humeral/femoral epiphyseal metric measurements of the fragmentary Malagasy subfossil lemur specimens were compared to the humeral/femoral epiphyseal metric measurements taken on complete Malagasy subfossil lemur specimens (for which a species-level taxonomic assignment was performed as detailed above). Fragmentary specimens were assigned to the same species of the complete specimen with the closest epiphyseal metric measurements. Data on epiphyseal metric measurements are in Supplementary Data 1-2.

3. µCT devices used to scan humeri and femora

- Phoenix | X-ray Nanotom, GE Sensing and Inspection Technologies GmbH
- XYLON FF35-CT-System, YXLON GmbH
- Microtomograph RX EasyTom 150
- Nikon XTH 225 ST
- GE v|tome|x

4. Slow arboreal mammal images used for Fig. 2

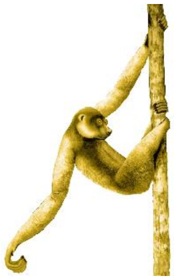	Modified from<br>https://commons.wikimedia.org/wiki/File:Palaeopropithecus_ingens.jpg<br><br>CC BY-SA 3.0, https://creativecommons.org/licenses/by-sa/3.0/deed.en, Life restoration of *Palaeopropithecus ingens.* Based on figure 6.2 and figure 6.6 of "Lemurs: Old and New" by E. L. Simons in Natural Change and Human Impact in Madagascar (Washington and London: Smithsonian Institution Press pp. 142-166), and correspondence with Dr. Laurie Godfrey.
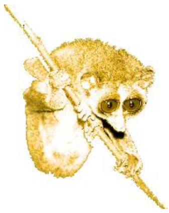	Modified from<br>https://en.wikipedia.org/wiki/Red_slender_loris#/media/File:Loris_tardigradus_tardigradus_001.jpg<br><br>CC BY-SA 4.0, https://creativecommons.org/licenses/by-sa/4.0/, picture uploaded by Dr. K.A.I. Nekaris on Wikipedia
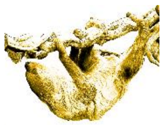	Modified from https://en.wikipedia.org/wiki/Two-toed_sloth#/media/File:Cholepus_didactylus_-_Flickr_-_Dick_Culbert.jpg<br><br>CC BY 2.0, https://creativecommons.org/licenses/by/2.0/deed.en, source: https://www.flickr.com/photos/92252798@N07/15238417480/; uploaded by: Dick Culbert from Gibsons, B.C., Canada (https://www.flickr.com/people/92252798@N07) on wikipedia
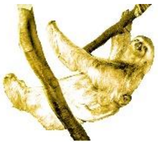	Modified from https://en.wikipedia.org/wiki/Pale-throated_sloth#/media/File:Bradypus_tridactylus.jpg<br><br>CC BY-SA 2.5 IT, https://creativecommons.org/licenses/by-sa/2.5/it/deed.en, The Natural history museum in Milan, Italy. Diorama with a *Bradypus tridactylus*. Picture by Giovanni Dall'Orto, April 22 2007, uploaded on Wikipedia.
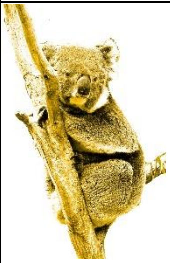	Modified from<br>https://en.wikipedia.org/wiki/File:Phascolarctos_cinereus_Bonorong.jpg<br><br>CC BY-SA 3.0, https://creativecommons.org/licenses/by-sa/3.0/deed.en, Koala (*Phascolarctos cinereus*), Bonorong Wildlife Park, Tasmania, Australia, Own work by JJ Harrison (https://www.jjharrison.com.au/)<br>Camera location: 42° 42′ 32.12″ S, 147° 16′ 17.55″ E, Kartographer map based on OpenStreetMap.

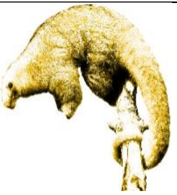	Modified from https://commons.wikimedia.org/wiki/File:Silky_Anteater.jpg<br><br>CC BY-SA 3.0, https://creativecommons.org/licenses/by-sa/3.0/deed.en, Date: 21 December 2013, 08:53:17, Own work by Quinten Questel, Camera location: 10° 27′ 40.04″ N, 61° 24′ 41.77″ W
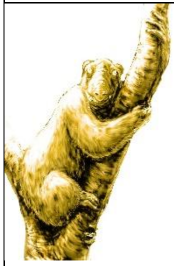	Modified from, https://commons.wikimedia.org/wiki/File:Megaladapis.jpg<br><br>CC BY-SA 3.0, https://creativecommons.org/licenses/by-sa/3.0/deed.en, *Megaladapis edwardsi*, Life restoration based on photos of skeletal remains and supported with correspondence with Dr. Laurie Godfrey, Date: 2010, Own work by FunkMonk (Michael B. H.)

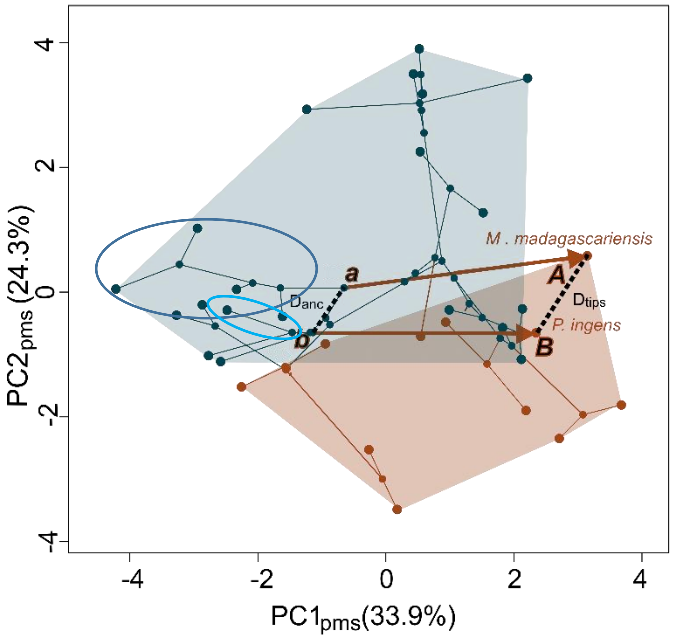

Supplementary Fig. 8. Phylomorphospace extracted and modified from Fig. 4 (panel c). The region of morphospace occupied by slow arboreal mammals, i.e. the hypothetic morphologically convergent taxa due to ecological convergence, is highlighted in light red. The region of morphospace occupied by non-slow arboreal mammals, closely related to the slow arboreal species but ecologically distinct, is highlighted in light blue. The evolutionary trajectories of the subfossil lemurs *Megaladapis madagascariensis* e *Palaeopropithecus ingens* are highlighted with red arrows. These two lineages independently (since they are not sister taxa) evolve toward the same region of the morphospace (i.e. they independently evolved a similar morphology). Differently from their ecologically distinct close relatives, lying within the 'non-slow arboreal' morphological variation (see dark blue and light blue ellipses), *M. madagascariensis* and *P. ingens* lie outside the 'non-slow arboreal' region, far from their close relatives (i.e. they show a different phenotype compared to the one of close relatives, that are subject to another functional regime) and they reached this condition starting from distant points in the phylogeny. This pattern potentially reflects similar phenotypic response to similar environmental pressures manifested at different points of the phylogenetic tree: i.e. the key concept of convergent evolution. To outline this scenario, we assessed the positions of focal, i.e. slow arboreal in this case, and non-focal, i.e. non-slow arboreal in this case, taxa and we added these evaluations to quantitative results from C indices. We believe that in this study this is the most suitable approach to detect convergence.

Under a strictly geometry-based definition of convergence (at the base of Grossnickle et al[11].'s Ct indices) these two taxa are not convergent. Indeed, two or more taxa are convergent if and only if their evolutionary trajectories are geometrically convergent, i.e. they tend to cross. If we assume as constant the extant phenotypes (represented by the positions **A** and **B**; along **AB** is measured the distance between extant taxa, D_{tips}, that is thus assumed constant too) the two trajectories may be convergent or not, depending on their reconstructed ancestral morphologies. The latter determines the position of ancestral nodes (**a** and **b**; along **ab** is measured the distance between the reconstructed ancestral phenotypes, D_{anc}). If we consider the simplified case of **ab** parallel to **AB:**

- If $D_{anc} > D_{tips}$ → convergent trajectories: convergent evolution, under a geometry-based concept
- If $D_{anc} < D_{tips}$ → divergent trajectories (see Figure): divergent evolution, under a geometry-based concept
- If $D_{anc} = D_{tips}$ → parallel trajectories: parallel evolution, under a geometry-based concept

Since D_{tip} is constant, D_{anc} alone determines the evolutionary process that we detect, but D_{anc} derives from ancestral states estimated assuming a random-walk process (Brownian Motion). Although it is true for C indices too (used in this work), we also relied on a qualitative assessment of pattern of focal vs. non-focal taxa on phylomorphospaces (see above), and not only on evolutionary trajectories orientations.

Supplementary References:

1. Alfieri, F., Botton-Divet, L., Nyakatura, J. A. & Amson, E. Integrative approach uncovers new patterns of ecomorphological convergence in slow arboreal xenarthrans. *J Mamm Evol* (2022) doi:10.1007/s10914-021-09590-5.
2. Bargo, M. S., Toledo, N. & Vizcaíno, S. F. Paleobiology of the Santacrucian Sloths and Anteaters (Xenarthra, Pilosa). in *In: Early Miocene Paleobiology in Patagonia. High Latitude Paleocommunities of the Santa Cruz Formation.* 216–242 (Vizcaíno S.F. Kay R.F., Bargo M.S. (eds) (doi: 10.1017/CBO9780511667381.014), 2012).
3. Varela, L., Tambusso, P. S., McDonald, H. G. & Fariña, R. A. Phylogeny, macroevolutionary trends and historical biogeography of sloths: insights from a Bayesian morphological clock analysis. *Syst. Biol.* **68**, 204–218 (2019).
4. Delsuc, F. *et al.* Ancient mitogenomes reveal the evolutionary history and biogeography of sloths. *Curr. Biol.* **29**, 2031-2042.e6 (2019).
5. Godfrey, L. R. *et al.* Limb joint surface areas and their ratios in Malagasy lemurs and other mammals. *Am J Phys Anthropol* **97 (1)**, 11–36 (1995).
6. Godfrey, L. R., Jungers, W. L., Wunderlich, R. E. & Richmond, B. G. Reappraisal of the postcranium of *Hadropithecus* (Primates, Indroidea). *Am J Phys Anthropol* **103**, 529–556 (1997).
7. Jungers, W. L. *et al.* Ecomorphology and Behavior of Giant Extinct Lemurs from Madagascar. in *In: Reconstructing Behavior in the Primate Fossil Record* 371–411 (Plavcan J.M., Kay R.F., Jungers W.L., van Schaik C.P. (doi: https://doi.org/10.1007/978-1-4615-1343-8_10), 2002).
8. Gommery, D., Ramanivosoa, B., Tombomiadana-Raveloson, S., Randrianantenaina, H. & Kerloc'h, P. Une nouvelle espèce de lémurien géant subfossile du Nord-Ouest de Madagascar (*Palaeopropithecus kelyus,* Primates). *Comptes Rendus Palevol* **8**, 471–480 (2009).
9. Goodman, S. M., Jungers, W. L. & Simeonovski, V. Plate 14: Anjohibe II—Inferences Based on Cave Remains and Aspects of the Organisms Living in the Adjacent Ecosystem. in *In: Extinct Madagascar: Picturing the Island's Past* 150–156 (2014).

10. Burney, D. A. *et al.* Subfossil lemur discoveries from the Beanka Protected Area in western Madagascar. *Quat. res.* **93**, 187–203 (2020).
11. Grossnickle, D. M. *et al.* Challenges and advances in methods for measuring phenotypic convergence. Pre-print. *bioRxiv* (2023) doi:https://doi.org/10.1101/2022.10.18.512739.
