# Supplementary material for: A macroevolutionary common-garden experiment reveals differentially evolvable bone organization levels in slow arboreal mammals
Source: Commun Biol. 2023 Sep 28;6:995. doi: 10.1038/s42003-023-05371-3 (PMC10539518; doi:10.1038/s42003-023-05371-3)
Supplement: Supplementary file 3 — Description of Additional Supplementary Data [file 42003_2023_5371_MOESM3_ESM.docx]

**Description of Additional Supplementary Files**

**File name:** Supplementary Data 1

**Description:** Data and information for the studied humeri. For each specimen, the dataset includes: · Taxon, collection and catalogue number (also detailing specimens included with isolated epiphyses, with ‘onlyProx’ and ‘onlyDist’ in the name) · Morphosource ARK ID code allowing to download the related virtual data; · Scanning resolution, i.e. voxel size, Resolution (mm); · Sizes of the proximal [(HeadPDLen(mm), HeadMLLen(mm), HeadAPLen(mm)] and distal [CapPDLen(mm), CapMLLen(mm), CapAPLen(mm) epiphyses · Cases of discarding from 3D GM analysis (DiscGM=1); · Cases of correction due to biased slices in CSP quantification (SliRemCSP=1); · Cases of discarding from CSP computation (DiscCSP=1); · Cases of necessity of extreme diaphyseal slices manual restoration followed by data interpolation (ExtSliRest=1); · Cases in which only the mid-diaphyseal level was analysable (Only50=1); · Cases in which only the mid-diaphyseal level was analysable but only following manual restoration (Rest50=1); · Cases of discarding of VOIs of trabecular bone (DiscProxTrab=1 and/or DiscDistTrab=1, for the proximal and distal epiphysis respectively); · Cases of restored VOIs of trabecular bone (RestProxTrab=1 and/or RestDistTrab=1, for the proximal and distal epiphysis respectively); · Diameters of trabecular VOIs [ROIproxDiam (mm) and ROIdistDiam (mm)]; · Relative resolution for each proximal and distal trabecular VOI (ROIproxRelRes and ROIdistRelRes); · Body mass proxy (BMproxy), computed as detailed in the text; · PC scores extracted from the 3D analysis (PC1-P91); · CSP raw data, extracted from both the 50% level (Parameter50) and averaging CSP along the diaphysis (ParameterAver) (ResC50 (%)- CSSAver); · Trabecular parameters raw data from each of the analyzed articular joint [DAprox- Av.Br.LenDist(mm)]

**File name:** Supplementary Data 2

**Description:** Data and information for the studied femora. For each specimen, the dataset includes: · Taxon, collection and catalogue number (also detailing specimens included with isolated epiphyses, with ‘onlyProx’ and ‘onlyDist’ in the name) · Morphosource ARK ID code allowing to download the related virtual data; · Scanning resolution, i.e. voxel size; Resolution(mm); · Sizes of the proximal epiphysis [(HeadPDLen(mm), HeadMLLen(mm), HeadAPLen(mm)], lateral condyle [LatConPDLen(mm), LatConMLLen(mm), LatConAPLen(mm)] and medial condyle[MedConPDLen(mm), MedConMLLen(mm), MedConAPLen(mm)] · Cases of discarding from 3D GM analysis (DiscGM=1); · Cases of correction due to biased slices in CSP quantification (SliRemCSP=1); · Cases of discarding from CSP computation (DiscCSP=1); · Cases of necessity of extreme diaphyseal slices manual restoration followed by data interpolation (ExtSliRest=1); · Cases in which only the mid-diaphyseal level was analysable (Only50=1); · Cases in which only the mid-diaphyseal level was analysable but only following manual restoration (Rest50=1); · Cases of discarding of VOIs of trabecular bone (DiscProxTrab=1, DiscLatConTrab=1 and DiscMedConTrab=1 fteral condyle and medial condyle respectively); · Cases of restored VOIs of trabecular bone (RestProxTrab=1, RestLatConTrab=1 and RestMedConTrab=1 for the proximal epiphysis, lateral condyle and medial condyle respectively); · Diameters of trabecular VOIs [ROIproxDiam (mm), ROILatConDiam (mm), ROIMedConDiam (mm)]; · Relative resolution for each proximal, lateral condyle and medial condyle trabecular VOI (ROIproxRelRes, ROILatConRelRes, ROIMedConRelRes); · Body mass proxy (BMproxy), computed as detailed in the text; · PC scores extracted from the 3D analysis (PC1-P91); · CSP raw data, extracted from both the 50% level (Parameter50) and averaging CSP along the diaphysis (ParameterAver) (ResC50 (%)- CSSAver); Trabecular parameters raw data from each of the analyzed articular joint [DAprox- Av.Br.MedCon(mm)]

**File name:** Supplementary Data 3

**Description:** time-calibrated tree used in the analysis

**File name:** Supplementary Data 4

**Description:** R code allowing to repeat statistical analyses
