# Supplementary material for: A macroevolutionary common-garden experiment reveals differentially evolvable bone organization levels in slow arboreal mammals
Source: Commun Biol. 2023 Sep 28;6:995. doi: 10.1038/s42003-023-05371-3 (PMC10539518; doi:10.1038/s42003-023-05371-3)
Supplement: Supplementary file 8 — Reporting Summary [file 42003_2023_5371_MOESM8_ESM.pdf]

nature portfolio

Corresponding author(s): Fabio Alfieri

Last updated by author(s): Sep 5, 2023

Reporting Summary

Nature Portfolio wishes to improve the reproducibility of the work that we publish. This form provides structure for consistency and transparency in reporting. For further information on Nature Portfolio policies, see our Editorial Policies and the Editorial Policy Checklist.

Statistics

For all statistical analyses, confirm that the following items are present in the figure legend, table legend, main text, or Methods section.

n/a	Confirmed	
☐	☒	The exact sample size (*n*) for each experimental group/condition, given as a discrete number and unit of measurement
☐	☒	A statement on whether measurements were taken from distinct samples or whether the same sample was measured repeatedly
☐	☒	The statistical test(s) used AND whether they are one- or two-sided<br>*Only common tests should be described solely by name; describe more complex techniques in the Methods section.*
☐	☒	A description of all covariates tested
☐	☒	A description of any assumptions or corrections, such as tests of normality and adjustment for multiple comparisons
☐	☒	A full description of the statistical parameters including central tendency (e.g. means) or other basic estimates (e.g. regression coefficient) AND variation (e.g. standard deviation) or associated estimates of uncertainty (e.g. confidence intervals)
☐	☒	For null hypothesis testing, the test statistic (e.g. *F*, *t*, *r*) with confidence intervals, effect sizes, degrees of freedom and *P* value noted<br>*Give P values as exact values whenever suitable.*
☒	☐	For Bayesian analysis, information on the choice of priors and Markov chain Monte Carlo settings
☒	☐	For hierarchical and complex designs, identification of the appropriate level for tests and full reporting of outcomes
☒	☐	Estimates of effect sizes (e.g. Cohen's *d*, Pearson's *r*), indicating how they were calculated

Our web collection on statistics for biologists contains articles on many of the points above.

Software and code

Policy information about availability of computer code

Data collection	We collected 109 humeri and 108 femora (Supplementary Data 1-2) from ten mammal museum collections in Austria, France, Germany, and the USA (Supplementary Note 1). Bones were scanned using micro-focus computed tomography (µCT) (Supplementary Note 3), generating image stacks (16-bit tifs). In VG Studio Max 3.3 (Volume Graphics, Heidelberg, Germany), each image stack was used to create a 3D mesh and was oriented. On each 3D mesh, we captured the shape through 3D GM locating anatomical landmarks + curve and surface sliding semilandmarks through MorphoDig and the R package 'Morpho'. In FIJI, on oriented stacks we measured cross-sectional properties, extracted Volumes of Interests (VOIs) of trabecular bone and we computed trabecular parameters on each VOI
Data analysis	Data were analysed in R version 4.1.2

For manuscripts utilizing custom algorithms or software that are central to the research but not yet described in published literature, software must be made available to editors and reviewers. We strongly encourage code deposition in a community repository (e.g. GitHub). See the Nature Portfolio guidelines for submitting code & software for further information.

Data

Policy information about availability of data

All manuscripts must include a data availability statement. This statement should provide the following information, where applicable:

- Accession codes, unique identifiers, or web links for publicly available datasets
- A description of any restrictions on data availability
- For clinical datasets or third party data, please ensure that the statement adheres to our policy

Raw data (Supplementary Data 1-2), time-tree (Supplementary Data 3) and Supplementary Information (including additional figures, tables and notes, besides credits and copyright licenses for slow arboreal mammal images used in Fig. 2), are available on Figshare (https://doi.org/10.6084/m9.figshare.22061207.v11). Image stacks and 3D meshes can be downloaded from MorphoSource (https://www.morphosource.org/projects/000393379, ARK ID codes in Supplementary Data 1-2), excluding those deriving from SMNS, Stuttgart, ZFMK, Bonn, ZSM, Munich, (all in Germany) and DPC, Duke University (USA) that are made available upon reasonable request.

Human research participants

Policy information about studies involving human research participants and Sex and Gender in Research.

Reporting on sex and gender	This study does not involve human research participants
Population characteristics	This study does not involve human research participants
Recruitment	This study does not involve human research participants
Ethics oversight	This study does not involve human research participants

Note that full information on the approval of the study protocol must also be provided in the manuscript.

Field-specific reporting

Please select the one below that is the best fit for your research. If you are not sure, read the appropriate sections before making your selection.

☐ Life sciences ☐ Behavioural & social sciences ☒ Ecological, evolutionary & environmental sciences

For a reference copy of the document with all sections, see nature.com/documents/nr-reporting-summary-flat.pdf

Ecological, evolutionary & environmental sciences study design

All studies must disclose on these points even when the disclosure is negative.

Study description	We compared convergence magnitude between external shape and internal structure of the humerus and the femur of convergently slow arboreal mammals. We measured external shape through high-density 3D geometric morphometrics and internal structure at different sub-regions (diaphysis with cross-sectional properties, epiphyses with trabecular parameters). We first identified traits significantly setting apart slow arboreal from non-slow arboreal species, through phylogenetically informed linear models and tests (i.e. Phylogenetic Generalized Least Square , PGLS, and Phylogenetic ANCOVAs). A body mass proxy was included as co-variate in each PGLS, after excluding potential interactions between body mass and the slow arboreal lifestyle. It allowed us to identify traits that are not only affected by slow arboreality but also by body size, and to use size-corrected values for these traits in following analyses. Size-correction was obtained taking the residuals of a linear regression between the trait and a body mass proxy. For each trait, the normality of residuals from PGLS was evaluated. If residuals clearly deviated from a normal distribution, the trait was natural log-transformed and the PGLS repeated. Traits significantly discriminating slow arboreal mammals were pooled by anatomical level and/or sub-region and further tested for convergence magnitude through the computation of C-indices of the 'convevol' R package. Moreover, convergence patterns were visualized through phylomorphospaces based on PCs deriving from Principal Component Analysis on humeral and femoral multivariate datasets including all traits significantly setting apart slow arboreal mammals.
Research sample	Measurements were taken from virtual data obtained micro CT-scanning 109 humeri and 108 femora of extant/extinct slow arboreal and non-slow arboreal mammals (Supplementary Table 1). All the bones, stored as dried preserved samples, were collected in museum collections (Museum für Naturkunde, Berlin, Germany, ZMB Mam; Staatliches Museum für Naturkunde, Stuttgart, Germany, SMNS; Zoologisches Forschungsmuseum Alexander Koenig, Bonn, Germany, ZFMK Mam; Zoologische Staatssammlung, Munich, Germany, ZSM; Naturhistorisches Museum, Wien, Austria, NMW; Muséum national d'Histoire naturelle, Paris, France, MNHN; American Museum of Natural History, New York, NY, USA, AMNH; Field Museum of Natural History, Chicago, IL, USA, FMNH; Yale Peabody Museum of Natural History, New Haven, CT, USA, YPM PU; Division of Fossil Primates, Duke Lemur Center, Durham, NC, USA; DCP). The specimens belong to organisms who died a long time ago, not at the request of our study. A part of the sample (i.e. xenarthrans) was already analyzed in Alfieri et al. 2022. Details on specimens characteristics are provided in Supplementary Data 1-2.
Sampling strategy	We studied a sample of mammal bone scans (i.e. 109 humeri + 108 femora of adult, wild-caught and non-pathological individuals)

Sampling strategy	representing 47 taxa. For each slow arboreal clade, the closely related non-slow arboreal taxa was included. It allows to detect convergence patterns, as planned. For each skeletal element we extracted data at several levels of bone organization (i.e. external shape, diaphyseal internal structure, epiphyseal internal structure), as required by our experimental question.
Data collection	Fabio Alfieri collected all the specimens in museums, following the aforementioned specimen inclusion criteria (also detailed in Supplementary Notes). No fieldwork and/or collection of novel biological/palaeontological material are involved in this study, since all the studied specimens are already collected and cured in museum collections. Fabio Alfieri and technicians of several institutions (detailed in Ackowledgements) digitised all the bones through micro CT-scanning. Fabio Alfieri collected morphological data on virtual datasets, with the help of Léo Botton-Divet and Jan Wölfer for external shape and the help of Eli Amson for internal bone structure.
Timing and spatial scale	Since skeletal specimens were taken from museum where they are collected, cured and made available to researchers, timing and spatial scale of data collection do not affect the work and its results
Data exclusions	Skeletal specimens not meeting the aforementioned inclusion criteria, were not chosen in museum collections. Some fragmentary/externally damaged specimens were discarded from external shape analysis. Some specimens showing a locally damaged/biased internal structure were discarded from internal structure analysis of the related sub-region. VOIs with less than 50 trabeculae were discarded. Details on discarded specimens and additional information on data exclusion are provided in Supplementary Data 1-2 and Supplementary Notes, respectively.
Reproducibility	All the studied specimens are identified with a catalogue number and the collection in which they are stored and cured (Supplementary Data 1-2). Moreover, virtual data deriving from each of them and analyzed in this work will be freely downloadable from MorphoSource (with the related ARK ID code listed in Supplementary Data 1-2), with only a minor part of the sample that, instead, will be made available upon reasonable request. Details on the procedures for morphological data extraction and statistical analysis are provided in Supplementary Information and the R code (Supplementary Data 4). All of this makes the entire study fully repeatable.
Randomization	This work focuses on species that are evolutionarily related, hence randomization is not applicable. We used phylogenetic comparative methods to account for non-independence of observations due to shared evolutionary history.
Blinding	Knowing which species is analyzed does not affect the results of this work, hence blinding was not relevant. We believe that the sample of species here studied is wide enough to detect the expected patterns.

Did the study involve field work? ☐ Yes ☒ No

Reporting for specific materials, systems and methods

We require information from authors about some types of materials, experimental systems and methods used in many studies. Here, indicate whether each material, system or method listed is relevant to your study. If you are not sure if a list item applies to your research, read the appropriate section before selecting a response.

Materials & experimental systems

n/a	Involved in the study
☒	☐ Antibodies
☒	☐ Eukaryotic cell lines
☐	☒ Palaeontology and archaeology
☒	☐ Animals and other organisms
☒	☐ Clinical data
☒	☐ Dual use research of concern

Methods

n/a	Involved in the study
☒	☐ ChIP-seq
☒	☐ Flow cytometry
☒	☐ MRI-based neuroimaging

Palaeontology and Archaeology

Specimen provenance	Details on fossil specimens provenance can be retrieved in the museum collections in which they are stored.
Specimen deposition	To the best of our knowledge, fossil specimens analyzed in this work (i.e. humeri and femora of Miocene Patagonian extinct sloths and Quaternary subfossil lemurs) are stored in the same collections from which we sampled them, i.e. Muséum national d'Histoire naturelle, Paris, France, MNHN; Field Museum of Natural History, Chicago, IL, USA, FMNH; Yale Peabody Museum of Natural History, New Haven, CT, USA, YPM PU; Division of Fossil Primates, Duke Lemur Center, Durham, NC, USA; DCP.
Dating methods	Dates were taken from previous studies

☐ Tick this box to confirm that the raw and calibrated dates are available in the paper or in Supplementary Information.

Ethics oversight	No fieldwork and/or collection of novel palaeontological material are involved in this study, since all the studied specimens are already collected and cured in museum collections.

Note that full information on the approval of the study protocol must also be provided in the manuscript.
